# Biofilm formation of *Staphylococcus aureus* on various implants used for surgical treatment of destructive spondylodiscitis

**DOI:** 10.1038/s41598-024-70244-6

**Published:** 2024-08-21

**Authors:** Amrei T. Zacher, Kamran Mirza, Lara Thieme, Sandor Nietzsche, Christian Senft, Falko Schwarz

**Affiliations:** 1 Department of Neurosurgery, Jena University Hospital, Friedrich-Schiller-University of Jena, Am Klinikum 1, 07747 Jena, Germany; 2 Institute of Infectious Diseases and Infection Control, Jena University Hospital, Friedrich-Schiller-University of Jena, Erlanger Allee 103, 07747 Jena, Germany; 3 Leibnitz Center for Photonics in Infection Research, Jena University Hospital, Friedrich- Schiller-University of Jena, Erlanger Allee 103, 07747 Jena, Germany; 4 Centre for Electron Microscopy, Jena University Hospital, Friedrich-Schiller-University of Jena, Ziegelmühlenweg 1, 07743 Jena, Germany

**Keywords:** Antimicrobials, Bacteria, Biofilms, Microbial communities, Diseases, Medical research, Neurology, Pathogenesis, Biomaterials, Diseases of the nervous system

## Abstract

The incidence of spondylodiscitis has witnessed a significant increase in recent decades. Surgical intervention becomes necessary in case of bone destruction to remove infected tissue and restore spinal stability, often involving the implantation of a cage. Despite appropriate treatment, relapses occur in up to 20 percent of cases, resulting in substantial economic and social burdens. The formation of biofilm has been identified as a major contributor to relapse development. Currently, there is no consensus among German-speaking spinal surgeons or in the existing literature regarding the preferred choice of material to minimize relapse rates. Thus, the objective of this study is to investigate whether certain materials used in spinal implants exhibit varying degrees of susceptibility to bacterial attachment, thereby providing valuable insights for improving treatment outcomes.Eight cages of each PEEK, titanium-coated PEEK (Ti-PEEK), titanium (Ti), polyetherketoneketone (PEKK), tantalum (Ta) and antibiotic-loaded bone cement were incubated with 20% human plasma for 24 h. Subsequently, four implants were incubated with *S. aureus* for 24 h or 48 h each. The biofilm was then removed by sonication and the attained solution plated for Colony Forming Units (CFU) counting. Scanning electron microscopy was used to confirm bacterial attachment. The CFUs have been compared directly and in relation to the cages surface area. The surface area of the implants was PEEK 557 mm^2^, Ti-PEEK 472 mm^2^, Ti 985 mm^2^, PEKK 594 mm^2^, Ta 706 mm^2^, bone cement 123 mm^2^. The mean CFU count per implant and per mm^2^ surface area after 24 h and after 48 h was calculated. Bone cement was found to have significantly more CFUs per mm^2^ surface area than the other materials tested. When comparing the CFU count per implant, bone cement was statistically significantly more prone to biofilm formation than PEEK after 48 h. There was no statistical significance between the other materials when comparing both CFU count per mm^2^ surface area and CFU count per implant. The electron microscopic analysis showed the attachment of the bacteria, as well as production of extracellular polymeric substances (EPS) as a sign for beginning biofilm formation. Antibiotic-loaded bone cement has shown statistically significantly more bacterial attachment than the other examined materials. No difference was found between the other materials regarding bacterial attachment after 24 h and 48 h. Proposed hypotheses for further studies include testing whether differences become apparent after longer incubation or with different pathogens involved in the pathogenesis of pyogenic spondylodiscitis.

## Introduction

Pyogenic spondylodiscitis is a disease of rising importance. In recent decades, the number of cases has increased continuously^1^. This trend can be attributed to the growing number of elderly, multimorbid and immunosuppressed patients. Additionally, the prevalence of risk factors such as intravenous drug abuse and spinal surgery has been on the rise^2,3^. Furthermore, advanced diagnostic measures have likely contributed to the observed increase in case numbers^2^. Simple pyogenic spondylodiscitis is initially treated conservatively with antibiotic therapy^2–8^. In many cases, surgery is required as the infection progresses^2,3,6^. Surgical therapy consists of debridement and resection of the destroyed disc and bone to eradicate the infection^8^. In cases of pronounced bone destruction, a cage or spinal implant may be used for spinal stabilization and compensation of height discrepancies^5,7^.

Approximately 20% of pyogenic spondylodiscitis cases experience relapse^9,10^. A key factor contributing to this is the formation of biofilm by residual bacteria on implanted cages^2,7,11–15^.

Bacterial biofilms consist of aggregated bacteria surrounded by a polymeric matrix and can attach to both biotic and abiotic surfaces^12,13,16–20^. Bacteria form these biofilm communities to protect themselves from environmental stressors, such as nutrient limitation or attacks by antibiotic therapy and the immune system^18,21,22^. Biofilms are highly tolerant to antibiotic therapy due to factors such as a downregulated metabolism, which shuts down the targets of many antibiotics, or a decrease in antibiotic penetration through the matrix^12,16–18^. Therefore, preventive strategies such as using anti-adhesive materials and implants to avoid bacterial attachment—the first step of the biofilm life cycle—are crucial to control recurrent biofilm-associated infections like spondylodiscitis^13,14,23^.

Overall, there is no common consensus on which material is more or less prone to biofilm formation and should therefore be used in surgical therapy of spondylodiscitis. There are many studies comparing materials regarding their susceptibility to bacterial adherence in retrospective or in vitro trials. However, most of them compare only two or three different materials to each other^10,24–32^. By comparing six of the most commonly used and new materials, polyetheretherketone (PEEK), titanium-coated PEEK (Ti-PEEK), titanium (Ti), tantalum (Ta), polyetherketoneketone (PEKK) and antibiotic-loaded bone cement, we hope to obtain a direct comparison of their susceptibility to biofilm formation. We chose *Staphylococcus aureus* for this experiment because it is the most common pathogen found in patients with pyogenic spondylodiscitis in Europe^6,7,11,33^.

The aim of this study is to prevent relapses after surgical resection and debridement of infected human tissue and bone. Therefore, we refer to the primary surgical situation. However, these materials can also be implanted in secondary revision surgery after removal of an infected implant.

We hypothesize that PEKK and Tantalum will demonstrate least bacterial attachment due to suspected intrinsic antimicrobial properties. Titan is well tested as an implant material, and we expect it to form little biofilm. We assume PEEK and Ti-PEEK to form more biofilm because of the cracks and holes on their surface in which bacteria can easily attach. We hypothesize antibiotic-loaded bone cement to be most prone to biofilm formation because of its rough surface structure and the presence of antimicrobials triggering biofilm mode of growth in pathogens. Bone cement is known to be susceptible to bacterial attachment^34^.

By conducting this comparative analysis, our objective is to provide evidence-based recommendations regarding the most appropriate material for surgical therapy of pyogenic spondylodiscitis, with the ultimate goal of reducing the risk of recurrence of infection.

## Material and methods

**Figure 1:**
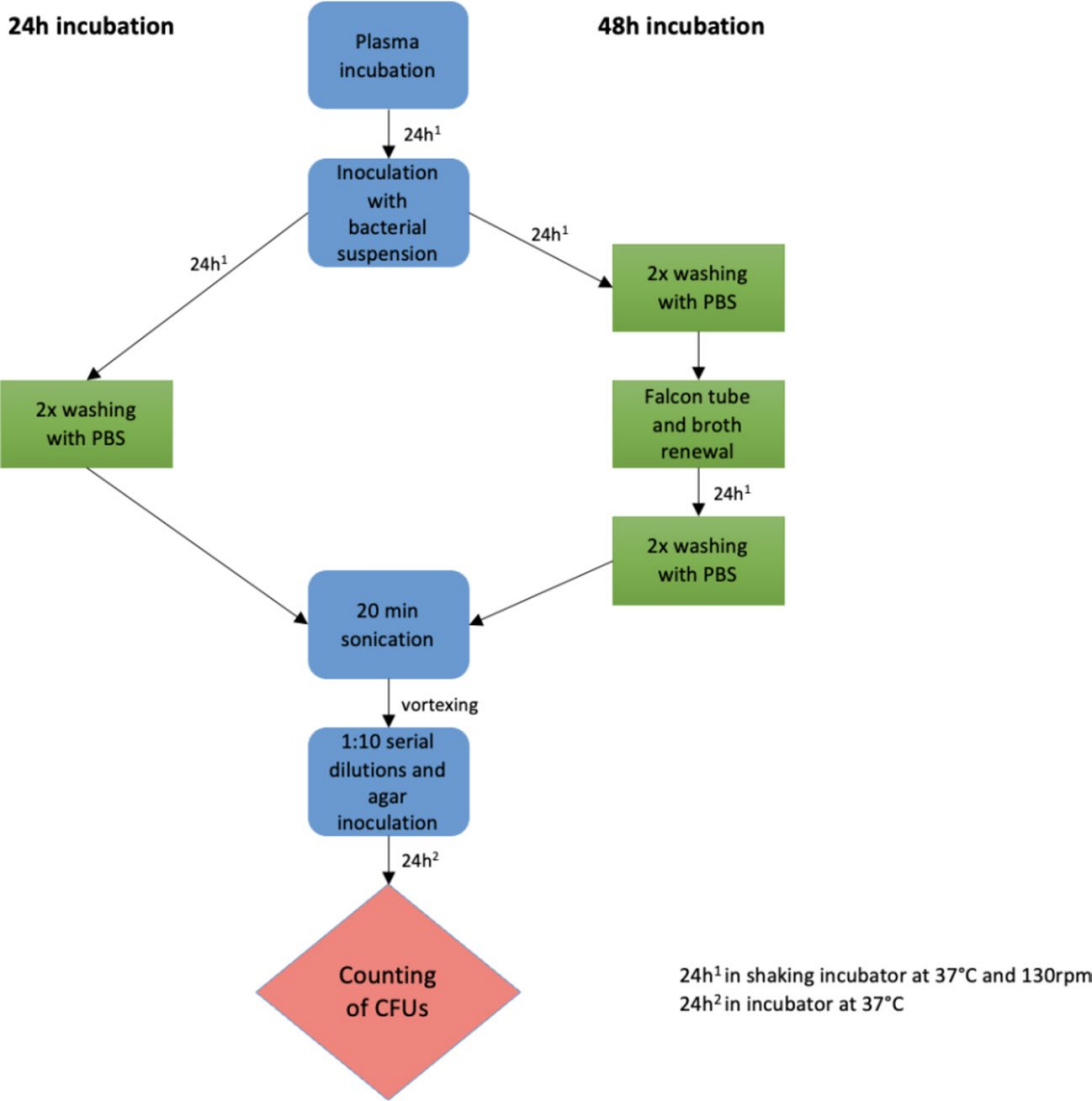
Flowchart presenting the experimental procedure to quantify the bacterial attachment for each implant.

The tested materials were PEEK, Ti-PEEK, Ti, Ta, PEKK and antibiotic-loaded bone cement. For each material, we incubated three cages for 24 h and 48 h each, with *S. aureus* and one cage as a negative control. After the incubation time, the cages were sonicated to dislodge the biofilm. Serial dilutions were made from the attained solution and cultures were plated. After 24 h, the Colony Forming Units (CFUs) were counted (see Fig. 1).

One PEEK cage was investigated with scanning electron microcopy (SEM). For this, it was cut in quarters. Two quarters went into the SEM directly after 48 h of incubation with *S. aureus*. One quarter was sonicated after 48 h of incubation before going into the SEM and the other quarter was used as negative control.

### Initial data collection

The materials used in the study were selected in reference to a survey conducted under German-speaking neurosurgeons as to which materials they prefer in surgical therapy of spondylodiscitis patients^35^.

### Implants

According to the answers to the survey, we gathered 8 cages each of PEEK Ayers Rock 5S (Spineway, 7 allée Moulin Berger, 69130 Ecully, FRANCE), titaniumcoated PEEK Mecta-C Interbody Fusion Cage 14mmx14mm 7° lordosis (Medacta Germany GmbH, Göppingen, Germany), Titanium Fortilink®-C IBF with TiPlus™ Technology (Surgalign, Wurmlingen, Germany), Tantalum TM-S Fusion Device Trabecular Metal^TM^ Technology 14x14mm, Angled, 5mm Height (Zimmer Biomet, Zug, Switzerland), PEKK Fortilink®-C IBF System with TETRAfuse® 3D Technology (Surgalign, Wurmlingen, Germany) and bone cement loaded with 0.5g Gentamicin and 2g Vancomycin COPAL® G+V (Heraeus Medical GmbH, Wehrheim, Germany). The cement was prepared according to the manufacturer’s instructions. When a liquid cement was obtained, it was formed under sterile conditions. After the cement had hardened, it was stored in a sterile plastic bag. Before usage for the experiment, it was autoclaved again.

All implants were similar in size to obtain comparable proportions. We acquired the total surface area of each implant (see Table 1). The surface areas of PEEK, Ti-PEEK, Titanium, Tantalum and PEKK were provided by the producing companies. The surface area of the bone cement cages was calculated after measuring them. After conducting the direct measurements, the average surface area of the bone cement cages was found to be 123 mm^2^, with a standard deviation of ± 16 mm^2^. The titanium cage had the largest surface area of 985 mm^2^ because it is manufactured with selective laser melting (SLM). Conversely, the bone cement cages demonstrated the smallest surface area.

**Table 1:** Total surface area in mm^2^ of the used implants.

Implants	Total surface area in mm^2^
PEEK	557
Ti-PEEK	472
Titanium	985
Tantalum	706
PEKK	594
Bone cement	123

### Plasma incubation

All cages were initially incubated with human plasma and Brain Heart infusion media (BHI). For every material, 5 ml of distilled water was injected in to citrated human plasma (Sigma-Aldrich, Taufkirchen, Germany) according to the instructions mentioned and agitated until the powder had dispersed completely. The resulting 5 ml plasma solution was then mixed with 15 ml BHI medium (Carl Roth GmbH + Co. KG, Karlsruhe, Germany). The implants were placed in a six-well plate (Greiner Bio-One GmbH, Frickenhausen, Germany) and 6.5 ml of the plasma-medium mix was added. The volume was enough to cover the implants with the plasma. The six-well plate was then incubated for 24 h at 37°C while shaking at 130 rpm.

### Preparation

To prepare the bacterial suspension, *S. aureus* DSMZ 28,763 strain was streaked on a blood agar plate (BD biosciences, Heidelberg, Germany) from the bacterial cryostock. The *S. aureus* DSMZ 28,763 is a standard laboratory strain from DSMZ-German Collection of Microorganisms and Cell Cultures GmbH (https://www.dsmz.de/collection/catalogue/details/culture/DSM-28763). The strain is a strong biofilm producer and has been used extensively for studying implant-associated biofilm in literature^36^. After 24 h incubation, five to six colonies were used to inoculate the BHI medium. The optical density of the inoculated medium was adjusted to 0.5 McFarland, corresponding to approximately 1.5 × 10^8^ CFU/mL, and used as the bacterial suspension for the implants.

### Biofilm formation

After coating the implants with plasma, three implants per material were transferred to the bacterial suspension of 0.5 McFarland in the 50 mL tubes and incubated for 24 h and 48 h each in a shaking incubator at 37 °C at 130 rpm. For the groups of 24 h and 48 h, there was one implant used for negative control each, which was placed in a tube filled with sterile BHI. For the implants with total incubation period of 48 h, the implants were removed from the bacterial suspension at 24 h, washed with 1 × Phosphate-buffered Saline (PBS) (Sigma-Aldrich, Taufkirchen, Germany) to remove any debris or unattached cells, transferred to fresh media and incubated for the remaining 24 h.

### Biofilm evaluation

After the incubation time, the implants were taken out of the tubes and washed twice with 1 × PBS to remove planktonic bacterial cells. The cages were sonicated in 1 × PBS for 20 min to detach the bacterial biofilm. Following this, a 1:10 serial dilution was performed, and the diluted samples were plated out on Müller-Hinton-agar plates (Oxoid, Thermo Fisher Scientific, Germany) with a spreader rod (Greiner Bio-One GmbH, Frickenhausen, Germany). After 24 h of incubation at 37 °C, the CFUs were counted (see Fig. 1). The calculation for the CFU/ implant was based on the whole volume of the 1 × PBS in which implants were sonicated and then further calculated into mm^2^/ implant.

### Scanning *electron* microscopy

A single PEEK cage was selected for investigation using SEM. For this purpose, it was cut in quarters with a slow speed saw IsoMet (Buehler, Esslingen, Germany) equipped with a diamond wafering blade and then autoclaved again to assure sterility. The PEEK cage was then subjected to the above protocol for 48 h incubation with one quarter as negative control. Two of the quarters were directly subjected to SEM analysis after 48 h of incubation. One quarter underwent sonication prior to SEM analysis. The samples were then placed each in a fixative solution (4% w/v paraformaldehyde, 2.5% v/v glutaraldehyde, 0.1 mol/L sodium-cacodylate buffer). After two hours at room temperature, samples were washed with 1 × PBS and stored in sodium-cacodylate buffer for SEM analysis.

### Statistical analysis

The raw data was processed using Excel. The data was then analyzed using GraphPad Prism 9. The mean and standard deviation were calculated for every group of material for 24 h and 48 h incubation. The evaluation for statistical significance was performed by ordinary one-way ANOVA. The confidence level for statistical significance was adapted for 95% (*p* < 0.05).

## Results

### Quantitative evaluation of bacterial attachment

**Figure 2:**
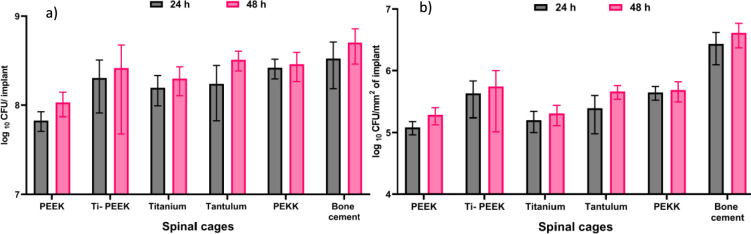
(**a**) Statistical mean CFU count/implant with standard deviation. (**b**) Statistical mean CFU count/mm^2^ surface area with standard deviation. For every material, three implants have been incubated for 24 h and 48 h each with *S. aureus*. After the incubation period, the cages have been sonicated. The attained solution was used for cultures. After 24 h, the CFUs have been counted.

Bacterial attachment took place on all materials (see Table 2 and Table 3). The quantitative analysis of CFU/implant showed the most attachment on antibiotic-loaded bone cement after both 24 h and 48 h. PEEK was least susceptible to bacterial attachment after 24 h and 48 h. After 24 h, Ti and Ta had more attachment than PEEK which was comparable to each other. Ti-PEEK and PEKK showed more attachment. After 48 h, the attachment had adjusted to similar CFUs on Ti, Ta, Ti-PEEK and PEKK. PEEK had the least bacterial attachment (see Fig. 2). Figure 2 shows the CFU/mm^2^ after 24 h and 48 h incubation. After 24 h, PEEK and Ti showed the least attachment, followed by Ta. Ti-PEEK and PEKK showed more, similar attachment. The results were similar after 48 h, only Ta has shown some more attachment after that time.

**Table 2:** CFU count per Implant after 24 h and 48 h of incubation with *S. aureus.*

Implants	Replicate 1	24 h eplicate 2	Replicate 3	Replicate 1	48 hReplicate 2	Replicate 3
PEEK	4.9 × 10^7^	7.4 × 10^7^	8.0 × 10^7^	1.3 × 10^8^	1.2 × 10^8^	7.0 × 10^7^
Ti-PEEK	3.3 × 10^8^	1.8 × 10^8^	9.1 × 10^7^	1.5 × 10^8^	5.1 × 10^8^	1.3 × 10^8^
Titanium	1.1 × 10^8^	2.2 × 10^8^	1.3 × 10^8^	1.7 × 10^8^	1.5 × 10^8^	2.8 × 10^8^
Tantalum	6.0 × 10^7^	2.7 × 10^8^	1.9 × 10^8^	2.3 × 10^8^	3.6 × 10^8^	3.8 × 10^8^
PEKK	1.9 × 10^8^	3.0 × 10^8^	3.0 × 10^8^	1.7 × 10^8^	3.8 × 10^8^	3.1 × 10^8^
Bonecement	5.0 × 10^8^	1.4 × 10^8^	3.5 × 10^8^	5.2 × 10^8^	7.1 × 10^8^	2.8 × 10^8^

**Table 3:** CFU count per surface area mm^2^ after 24 h and 48 h of incubation with *S. aureus.*

Implants	Replicate 1	24 h Replicate 2	Replicate 3	Replicate 1	48 h Replicate 2	Replicate 3
*PEEK*	8.8 × 10^4^	1.3 × 10^5^	1.4 × 10^5^	7.0 × 10^5^	2.1 × 10^5^	1.3 × 10^5^
*Ti-PEEK*	7.0 × 10^5^	3.9 × 10^5^	1.9 × 10^5^	3.1 × 10^5^	1.1 × 10^6^	2.8 × 10^5^
*Titanium*	1.1 × 10^5^	2.3 × 10^5^	1.4 × 10^5^	1.7 × 10^5^	1.5 × 10^5^	2.8 × 10^5^
*Tantalum*	8.5 × 10^4^	3.8 × 10^5^	2.7 × 10^5^	3.3 × 10^5^	5.1 × 10^5^	5.4 × 10^5^
*PEKK*	3.1 × 10^5^	5.0 × 10^5^	5.1 × 10^5^	2.9 × 10^5^	6.4 × 10^5^	5.2 × 10^5^
*Bonecement*	4.1 × 10^6^	1.2 × 10^6^	2.8 × 10^6^	4.2 × 10^6^	5.8 × 10^6^	2.3 × 10^6^

Antibiotic-loaded bone cement showed to have grown significantly more CFU/implant after 48 h of incubation compared to PEEK 48 h. There were no statistical significances when comparing the CFUs/implant of the other materials. When comparing the CFU/mm^2^ surface area, antibiotic-loaded bone cement displayed significantly more bacterial attachment than all the other materials after both 24 h and 48 h. All the other materials did not show any statistically significant differences in biofilm formation when CFU/mm^2^ surface area were compared to each other (see Table 4).

**Table 4:** Values of CFU/implant and CFU/mm^2^ surface area of materials compared to each other.

Group	Compared Implants	Significance *p* < 0.05 (*), *p* < 0.01 (**), *p* < 0.001 (***), *p* < 0.0001 (****)
CFU/implant	PEEK 48 h vs. bone cement 48 h	*
CFU/mm^2^ surface area	PEEK 24 h vs. bone cement 24 h	**
	PEEK 48 h vs. bone cement 48 h	****
	Ti-PEEK 24 h vs. bone cement 24 h	*
	Ti-PEEK 48 h vs. bone cement 48 h	****
	Ti 24 h vs. bone cement 24 h	**
	Ti 48 h vs. bone cement 48 h	****
	Ta 24 h vs. bone cement 24 h	**
	Ta 48 h vs. bone cement 48 h	****
	PEKK 24 h vs. bone cement 24 h	*
	PEKK 48 h vs. bone cement 48 h	****

The results were analyzed for statistical significance using ordinary one-way ANOVA with GraphPad Prism. A *p* value of < 0.05 was deemed statistically significant. Only the statistically significant results are presented. The statistical analyses comparing the other implants did not show statistical significance (*p* > 0.05). Qualitative evaluation of bacterial attachment.

SEM imaging of a PEEK cage was done to visualize the formation of a biofilm on the implant.

**Figure 3:**
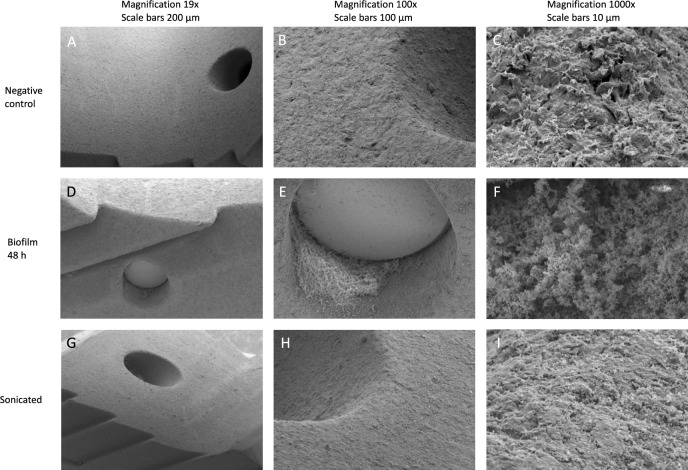
Scanning electron microscopic images of the surface of a PEEK spinal cage. (**A**–**B**) and (**C**) after initial coating with human plasma and incubation with pure medium (see text for details). (**D**–**F**) after 48 h incubation with *S. aureus* suspension. (**G**–**I**) after sonication post 48 h incubation with *S. aureus* suspension.

**Figure 4:**
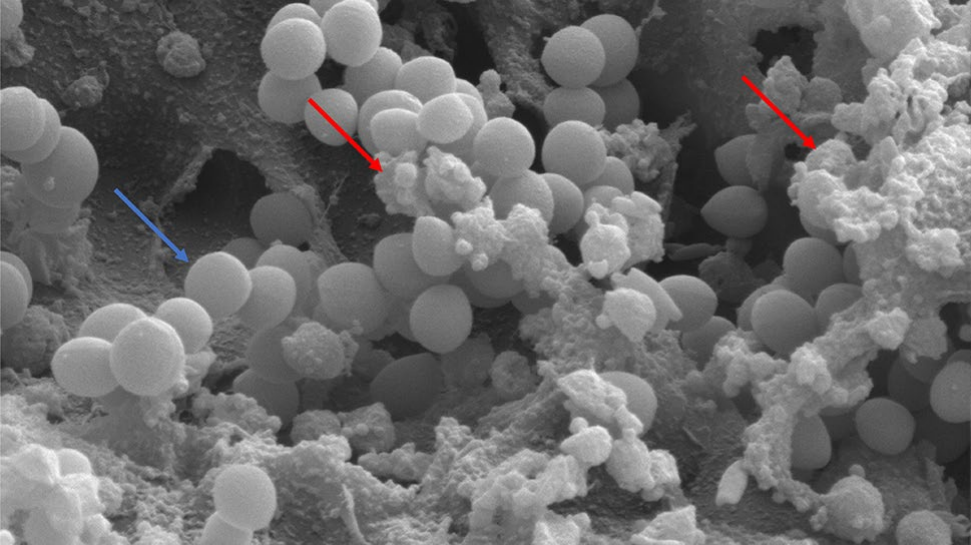
Scanning electron microscopic images of the surface of a PEEK spinal cage after initial coating with human plasma (see text for details) and 48 h of incubation with *S. aureus* suspension. Some exemplary *S. aureus* bacteria are marked with a blue dart. Between them, EPS can be seen as marked with red darts. The beginning of EPS production marks the initial stage of biofilm formation and shows that bacterial adhesion has taken place. Magnification 10.000x, scale bars 1 µm.

Figure 3 shows the surface of a PEEK cage with plasma coating as negative control, after 48 h of incubation with *S. aureus* and after sonication. The negative control shows the plasma coating on the surface in absence of bacteria. After 48 h of incubation, this surface is covered by multiple *S. aureus* bacteria. Bacterial attachment is observed in even higher extent in the edges and holes of the implant. With higher magnification, the extracellular polymeric substance (EPS) between the bacteria can be observed (see Fig. 4). After sonication, most of the bacteria are removed. However, some remain on the surface.

## Discussion

Spondylodiscitis is a disease with rising incidence in Europe^1^ that comes with enormous medical, social and economic costs^8^. The risk of infection relapse significantly contributes to these costs^11,14^, with biofilm formation on inserted implants being implicated as a key factor^2,7,11–14^.

Determining the optimal material for cage insertion in spondylodiscitis surgery to prevent relapses remains an unanswered question in the literature. Ideally, the chosen material should promote osseointegration while inhibiting bacterial attachment^13,14,22,23,37–41^. However, finding a material that possesses both properties proves to be a challenge. In fact, many companies producing spinal cages even name active infection in the surgical site as a contraindication for implantation. Nevertheless, in many cases, a cage must be placed to compensate height difference and avoid spinal deformities^5,7^. Over the years, many in vitro or retrospective studies have been conducted to find out if any material proves to be superior to the other. However, most of these studies compared only two materials to each other. Many of the results are contradictory or do not find any difference between the effects on bacterial attachment and biofilm formation at all^10,24–32^.

To gain a broader view, we compared all of the most used materials to each other.

There was a statistically significant difference between the bacterial attachment per implant on antibiotic-loaded bone cement compared to PEEK after 48 h of incubation. When comparing the CFUs per mm^2^ surface area, antibiotic-loaded bone cement showed to be significantly more prone to biofilm formation than any of the other tested materials after both 24 h and 48 h.

The SEM pictures show plasma on the surface of the depicted cage as well as bacterial microcolonies and EPS. The presence of self-produced EPS is an important characteristic of bacterial biofilms^13,20,22^. Because of this, we can conclude that bacterial attachment has indeed taken place during the experiment and that biofilm formation has started.

In order to mimic one aspect of the in vivo conditions, human plasma was utilized^42^. During surgery to implant a cage, blood components and cellular fluids are always present, coating the implant with host proteins as it is brought in place^40^. This serves as a conditioning film that is known to promote bacterial attachment and biofilm formation^43,44^. Notably, direct interactions between bacterial adhesins and specific human plasma proteins have been demonstrated to mediate bacterial adhesion^39^, specifically including the case of *S. aureus*^42,45^.

Based on the SEM images of the PEEK cage, we proved that bacterial attachment has indeed taken place on this specific implant. We hypothesize that this has taken place on the other materials as well since all of them underwent the same protocol. However, we cannot prove this since a lack of technical possibilities and resources prevented SEM imaging of all the tested materials, providing a limitation to this study.

One limitation of this study is that it was conducted through in vitro experimentation. Therefore, while these results provide valuable insights, they cannot be directly extrapolated to the clinical setting. One important factor that influences the in vivo scenario, but cannot be assessed in this in vitro study, is the direct interaction between the implant material and host cells. Previous indications suggest that Tantalum may have a direct enhancing effect on host immune cells through leukocyte activation, which play a role in intrinsic antibacterial activity^46^. However, other studies could not reproduce this finding^47^. Additionally, Tantalum is suspected to induce stem cells, thereby promoting osseointegration^27^.

Another example of supposedly intrinsic antibiofilm characteristics is PEKK which has been found to reduce bacterial and increase osteoblast attachment. It is hypothesized that this might come from the nanopatterned surface, which is not specific for PEKK but can also be found on different materials, for example Titanium surfaces with nanotubular structures^48^.

It has been reported that when an implant is brought into the body, a “race” between attachment of osteoblasts and pathogens occurs^49^. Whoever attaches first leaves less space for the attachment of the other. By these means, it is important to discuss the surface roughness of the implants. Surface roughness is associated with higher susceptibility to bacterial attachment, since it comes with a greater overall surface area and provides more edges and pores for bacteria to hide^10,25,50^. However, it also improves attachment of osteoblasts out of the same reasons^37,51^. From a microbiological view, the implants surface should be as smooth as possible. From an osseointegration point of view on the other hand, the surface should be as porous as possible^22^.

The surface energy, which is increased by hydrophily/high wettability, enhances the attachment of osteoblasts^38,40^ and therefore leaves less place for bacterial colonization. Even though surface energy and other surface properties play a role too, surface roughness is suspected to have the major effect on biofilm formation^25^.

Paradoxically, the more the surface roughness increases from micro to nano roughness, the more it acts antimicrobial while promoting osseointegration^38^. There had been prior descriptions of this especially for PEKK as mentioned earlier, but also for nano surfaced Titanium^28,48^. The nano columns on its surface are said to alter initial protein interactions between the implant and the host cells, subsequently improving osteoblast adhesion and osseointegration^52,53^. At the same time, their sharp-edged tips both limit anchoring points for pathogens on their surface as well as rupturing bacterial walls, actively killing bacteria^23,38,54^.

Moreover, the in vivo situation provides bacteria with a current source of nutrients. Even though we tried to mimic this feature by changing the media after 24 h for the 48 h incubation, we cannot completely simulate this. This might also be a reason for the stagnation in bacterial growth between 24 and 48 h. If nutrients are exhausted, bacteria detach from the biofilm to become planktonic again, seeking a better habitat rather than further contributing to biofilm growth^20,21,44^. Since the incubated implants have been washed with PBS before sonication, only the bacteria within the biofilm were detected in the CFU cultures.

On the other hand, it is known that cells within a biofilm are generally most active within the first phases of biofilm development while activity decreases after primary attachment^12^.

Biofilm formation is a mechanism of defense, for example against attack by antibiotics or the host immune cells^15,21^. Since there was no antimicrobial agent in the media or anywhere in the experiment, the stagnation of growth could also show the lack of need for biofilm formation. The bacteria could become planktonic again without consequences. However, bone cement was the only material in which antimicrobial agents have been present. This might have exerted selective pressure on the bacteria to go into biofilm mode of growth, explaining the extensive bacterial attachment on this material compared to the other materials. When bone cement is loaded with antibiotics, the release of the antimicrobial agent happens in a burst, killing most of the susceptible bacteria^34,55^. The initial burst is followed by release of subtherapeutic doses^34,56^. This puts the remnant bacteria under selective pressure and triggers biofilm mode of growth^56^. The induction of small colony variants (SCV) and hence biofilm formation of *S. aureus* strains has been shown for both vancomycin^56^ and gentamicin^57^ specifically. Additionally, as the antibiotic is released from the bone cement, drug levels in the surrounding tissue fall, which results in the surface becoming susceptible to bacterial attachment^34^.The inherent characteristics of bone cement make the decrease in antibiotic release and resulting subinhibitory concentrations unavoidable^34^, presenting a disadvantage of antibiotic-loaded bone cement. Exposing bacteria to subtherapeutic doses of antibiotics also promotes the development of genetic alterations and resistant bacterial phenotypes, posing an additional risk in healthcare^34^.

The exact concentration of Vancomycin and Gentamicin in the cages is not known. The used package included 40 g of powder containing 0.5 g Gentamicin and 2 g Vancomycin. Out of the package, 15 cages have been formed, resulting in an average concentration of 0.03 g/25 mg Gentamicin and 0.13 g/125 mg Vancomycin per cage. However, there are concerns about the dosage of antibiotics in bone cement implants. The cages are hand-mixed from formulations in the operating room by the surgeon without standardization^34^. This leads to differing concentrations in the individual cages. Since the single implants were not tested in a laboratory, their exact antibiotic concentration is unknown. This poses another limitation to the study. However, the cages were prepared the way that they are modeled in everyday medical practice, in which there is no testing of the implanted cages either. Hence, the differing antibiotic concentrations in bone cement implants pose a general disadvantage to their use.

Plasma further enhances the risk for biofilm formation. It inhibits the effectiveness of the initial burst release, serving as a physical barrier between released antibiotics and bacterial colonies. Plasma coating has been shown to specifically increase tolerance of *S. aureus* strains to vancomycin in vitro^58^. In addition, plasma generally enhances bacterial attachment due to protein interactions as discussed above^43,44,58^. These factors play a role in the development of the extensive amount of biofilm found on antibiotic loaded bone cement in this study. Other similar studies without plasma incubation of the implants showed strong bactericidal effects of antibiotic loaded bone cement even against Methicillin-resistant *Staphylococcus aureus* (MRSA)^59^. However, as discussed above, the attachment of plasma on the implants surface plays a crucial role in the in vivo situation^40,42,58^.

Nonetheless, most studies examining biofilm formation on implant materials are exerted without plasma coating^59^. We propose the use of plasma in future experiments to mimic the in vivo situation more accurately.

This study could only detect biofilm through the bacteria removed from the surface by sonication. Yet, as seen in the SEM images, not all pathogens are cleared by sonication. It is possible that this influenced the measured CFUs. Especially in case of very porous materials, the ultrasound might not penetrate through the whole implant. This is supported by the SEM images, in which remnant bacteria were located particularly in edges and holes of the implant (see Fig. 3).

We used *S. aureus* as the pathogen in this study since it is the most common bacteria causing pyogenic spondylodiscitis in Europe^6,7,11,33^. Other bacteria are also regularly found as cause of spondylodiscitis in patients such as *E. coli*, different *Staphylococci* and *Streptococci* and others^2,7^. It has been shown that different bacteria react differently to different materials. One of many examples is that nano surfaces might have differing effects on gram positive and gram negative bacteria^28,38,54^. This was found for other materials and bacterial strains as well^39,42^. Accordingly, the results of this experiment cannot be transferred to any bacterial strain. Further studies need to be carried out with different pathogens to see if they might react differently to the materials.

To compensate the different cage sizes, we chose to not only analyze the CFUs per implant but also the CFUs per mm^2^ surface area.

Another limitation is the implants short incubation period. In the future, similar experiments should be conducted to see how the biofilms develop over a longer duration of time.

## Conclusion

Biofilm infections are associated with complications, reoperations, and high social and medical costs. It is important to find a way to prevent the formation of biofilms and their consequences.

We have shown that antibiotic-loaded bone cement is significantly more prone to biofilm formation when compared to PEEK, Ti-PEEK, Titanium, Tantalum and PEKK. We did not find a statistically significant difference regarding bacterial attachment between the other tested materials. According to this, we would discourage from using antibiotic-loaded bone cement in spondylodiscitis surgery.

We have found a stagnation in biofilm growth when comparing 24 h and 48 h incubation period. This is likely caused by decreased activity after initial attachment and further provoked by a lack of triggers for further biofilm formation in the in vitro situation, namely providing a current source of nutrients and the absence of antimicrobial agents.

It has been shown that plasma promotes the attachment of bacteria in different ways, for example through direct protein interactions between plasma and bacteria, by serving as a physical barrier between antimicrobials and pathogens and promoting antibiotic resistance.

Further studies should be done with longer incubation periods over several days or weeks, to examine how the attachment develops into a full-grown biofilm. Studies with the other common pathogens involved in spondylodiscitis would also be of interest, for example *E. coli* or *S. epidermidis*, to understand if they interact differently with the materials and if certain pathogens indicate the use of specific materials.

Moreover, to get a better image on the effects that play a role in vivo, clinical trials or the analyzation of retrospective data of patients after spondylodiscitis surgery could be useful.

## Humans and animal resources

The authors state that no humans or animals were involved in the study. The raw datasets used and analyzed for this study are available from the corresponding author upon reasonable request. Three of the authors involved are neurosurgeons.

## Data Availability

The raw datasets used and analyzed for this study are available from the corresponding author upon reasonable request.
